# Supplementary material for: Accurate detection of paroxysmal atrial fibrillation with certified-GAN and neural architecture search
Source: Sci Rep. 2023 Jul 14;13:11378. doi: 10.1038/s41598-023-38541-8 (PMC10349064; doi:10.1038/s41598-023-38541-8)
Supplement: Supplementary file 1 — Supplementary Information. [file 41598_2023_38541_MOESM1_ESM.pdf]

A Supplementary materials

A.1 Reproducibility Checklist

1. For all authors...
   (a) Do the main claims made in the abstract and introduction accurately reflect the paper's contributions and scope? [Yes]
   (b) Did you describe the limitations of your work? [Yes] We discuss limitations of this work in Section 7
   (c) Did you discuss any potential negative social impacts of your work? [Yes] We discuss any societal impacts of this work in Section 7
2. If you are including theoretical results...
   (a) Did you state the full set of assumptions of all theoretical results? [Yes] All assumptions are described in the paper as well as the detail in the Appendix section.
   (b) Did you include complete proofs of all theoretical results? [N/A]
3. If you ran experiments...
   (a) Did you include the code, data, and instructions needed to reproduce the main experimental results, including all requirements (e.g., `requirements.txt` with explicit version), an instructive `README` with installation, and execution commands (either in the supplemental material or as a URL)? [Yes] Added all required hyper-parameters (Section 5), seeds, download links to databases, and GitHub repository.
   (b) Did you include the license of the datasets? [N/A] Our experiments were conducted on publicly available datasets and we have not introduced new datasets.
   (c) Did you include the raw results of running the given instructions on the given code and data? [Yes] All results are using the provided code.
   (d) Did you include scripts and commands that can be used to generate the figures and tables in your paper based on the raw results of the code, data, and instructions given? [Yes] See Code ReadMe file.
   (e) Did you ensure sufficient code quality such that your code can be safely executed and the code is properly documented? [Yes]
   (f) Did you specify all the training details (e.g., data splits, pre-processing, search spaces, fixed hyperparameter settings, and how they were chosen)? [Yes] For our experiments, we used the PhysioNet PxAF prediction challenge database (download link). Plus, all details are explained in Section 5 and Supplementary.
   (g) Did you ensure that you compared different methods (including your own) exactly on the same benchmarks, including the same datasets, search space, code for training and hyperparameters for that code? [Yes] Please see Section 6.2
   (h) Did you run ablation studies to assess the impact of different components of your approach? [Yes] Section 6.2 compares the classification results of the proposed method on three different datasets, including $D_{Original}$, D_{GAN}, and D_{CGAN}.
   (i) Did you use the same evaluation protocol for the methods being compared? [Yes]
   (j) Did you compare performance over time? [Yes] Anytime performance was assessed with the number of GPU hours as explained in Section 5.2.
   (k) Did you perform multiple runs of your experiments and report random seeds? [Yes] We reran the Deep-PxAF search procedure three more times with different random seeds to verify the reproducibility of the results. Results show that the average of multiple runs converges to neural architectures with similar results with the standard deviation (STD) of 0.2% for Deep-PxAF trained on D_{CGAN}. [Yes] Please check Table 5.
   (l) Did you include the total amount of compute and the type of resources used (e.g., type of GPUs, internal cluster, or cloud provider)? [Yes]

(m) Did you report how you tuned hyperparameters, and what time and resources this required (if they were not automatically tuned by your AutoML method, e.g. in a NAS approach; and also hyperparameters of your own method)? [N/A] In this paper, we did not use any method for optimizing learning hyperparameters. for the NAS, we use the DARTS method with default hyperparameters (please see Section 4.4).

A.2 Qualitative Analysis of the Searched Cells

Fig. 10 depicts the best cells searched by Deep-PxAF for the D_{CGAN} database. For the normal cell, DARTS tends to increase the portion of dilated convolution separable convolution (`sep_conv`) operations with the 5×5 kernel size. This is because larger kernel sizes (5×5) improve the representational power of the network. In contrast, the reduction cell has many average pooling operations for compressing the information across the spatial dimension. This is because pooling operations can increase the nonlinear representation ability of the network. Referring to the recurrence graphs in which rhythmic contents of ECG are preserved within the squares of 4 second (see Fig2, one can intuitively understand that an optimal kernel size is one that can include rhythms. A small kernel size can negatively impact the learning quality due to its failure to incorporate rhythmic content.

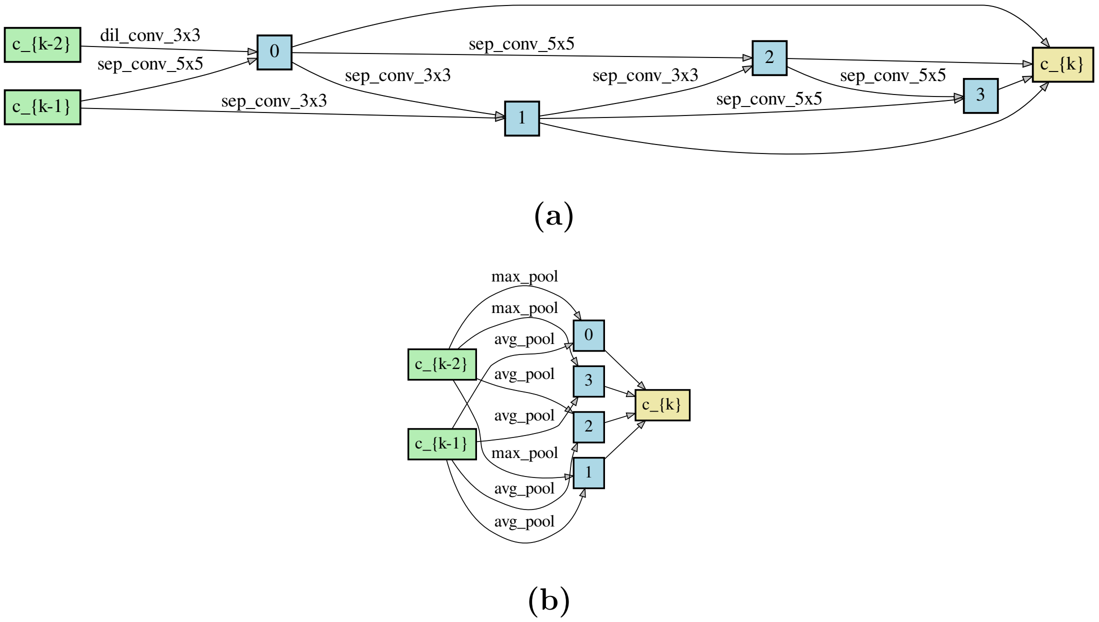

Figure 10: (a) Normal cell. (b) Reduction cell.

A.3 Details of Comparison Baselines

1. **Pourbabaee et al. [48]:** To obtain the optimal classification accuracy, a three-layer CNN architecture including convolutional, sub-sampling, and K-nearest neighbor (KNN) layers are utilized in which the optimal network parameters have been represented in Table 6.

2. **Surucu et al. [55]:** To achieve the best classification performance, a six-layer CNN architecture including three one-dimensional convolutional, dropout, pooling, and two fully-connected layers are utilized.

Table 6: The configuration setup of the PxAF detection method proposed by Pourbabaee et al. [48].

**Parameter**	**Value**
# Epochs	88
Optimizer	SGD
Learning Rate (lr)	0.09
Momentum	0.9
Sub-sampling Layer Kernel Size	128
# KNN Clusters	2
